# Delivering health promotion during school closures in public health emergencies: building consensus among Canadian experts

**DOI:** 10.1093/heapro/daad172

**Published:** 2023-12-13

**Authors:** Julia Dabravolskaj, Paul J Veugelers, Boshra A Mandour, Jenn Flynn, Katerina Maximova

**Affiliations:** MAP Centre for Urban Health Solutions, Li Ka Shing Knowledge Institute, St. Michael’s Hospital, 209 Victoria St, Toronto, ON M5B 1T8, Canada; Dalla Lana School of Public Health, University of Toronto, 155 College St Room 500, Toronto, ON M5T 3M7, Canada; School of Public Health, University of Alberta, 3-50E University Terrace, 8303 112 Street NW, Edmonton, AB T6G 1K4, Canada; School of Public Health, University of Alberta, 3-50E University Terrace, 8303 112 Street NW, Edmonton, AB T6G 1K4, Canada; APPLE Schools, 206-A, 12227 – 107 Ave NW, Edmonton, AB T5M 1Y9, Canada; MAP Centre for Urban Health Solutions, Li Ka Shing Knowledge Institute, St. Michael’s Hospital, 209 Victoria St, Toronto, ON M5B 1T8, Canada; Dalla Lana School of Public Health, University of Toronto, 155 College St Room 500, Toronto, ON M5T 3M7, Canada

**Keywords:** public health emergencies, COVID-19 school closures, health promotion practices, Delphi survey, school emergency preparedness plans

## Abstract

School-based health promotion is drastically disrupted by school closures during public health emergencies or natural disasters. Climate change will likely accelerate the frequency of these events and hence school closures. We identified innovative health promotion practices delivered during COVID-19 school closures and sought consensus among education experts on their future utility. Fifteen health promotion practices delivered in 87 schools across Alberta, Canada during COVID-19 school closures in Spring 2020, were grouped into: ‘awareness of healthy lifestyle behaviours and mental wellness’, ‘virtual events’, ‘tangible supports’ and ‘school-student-family connectedness’. Two expert panels (23 school-level practitioners and 20 decision-makers at the school board and provincial levels) rated practices on feasibility, acceptability, reach, effectiveness, cost-effectiveness and other criteria in three rounds of online Delphi surveys. Consensus was reached if 70% or more participants (strongly) agreed with a statement, (strongly) disagreed or neither. Participants agreed all practices require planning, preparation and training before implementation and additional staff time and most require external support or partnerships. Participants rated ‘awareness of healthy lifestyle behaviours and mental wellness’ and ‘virtual events’ as easy and quick to implement, effective and cost-effective, sustainable, easy to integrate into curriculum, well received by students and teachers, benefit school culture and require no additional funding/resources. ‘Tangible supports’ (equipment, food) and ‘school-student-family connectedness’ were rated as most likely to reach vulnerable students and families. Health promotion practices presented herein can inform emergency preparedness plans and are critical to ensuring health remains a priority during public health emergencies and natural disasters.

**Contribution to Health Promotion**

- This study sought consensus among education experts about innovative health promotion practices delivered during COVID-19 school closures in Alberta, Canada.
- These practices can inform emergency preparedness plans for school closures due to public health emergencies and natural disasters.
- Practices focusing on raising awareness of healthy lifestyle behaviours and mental wellness and organizing virtual events were rated as easy and quick to implement, effective, cost-effective, sustainable, easy to integrate into curriculum, well received by students and teachers and require no additional funding/resources.
- Providing tangible supports and focusing on the school–student–family connectedness can reach vulnerable students and families.

## BACKGROUND

Chronic or non-communicable diseases (NCDs) and mental disorders are highly common, regardless of age, culture, race/ethnicity, gender or income, and their prevalence continues to rise, particularly among those living in socioeconomically disadvantaged households and communities (Hanson and Gluckman, 2011; World Health Organization, 2022a). While NCDs are the leading cause of death, accounting for 74% of deaths globally (World Health Organization, 2022b), mental disorders (i.e. depressive and anxiety disorders, bipolar disorder, schizophrenia, autism spectrum disorders, eating disorders, among others) are the leading cause of disability worldwide (GBD 2019 Mental Disorders Collaborators, 2022). NCDs and some mental disorders can be prevented through improvements in physical activity, diet and other lifestyle behaviours, which, once established, are very difficult to reverse (Lake *et al*., 2006; Telama, 2009; Biddle *et al*., 2010). To curb the burden of NCDs and mental illness, health promotion interventions should be implemented early in life, during childhood and adolescence. Health promotion interventions delivered in the school setting are particularly promising (Pulimeno *et al*., 2020), as they have been shown to be effective (Dabravolskaj *et al.*, 2020) and cost-effective, have high return-on-investment (Ekwaru *et al.*, 2021), and reduce health inequalities (Moor *et al*., 2022). Foundational to school-based health promotion, the Health Promoting School (HPS) approach underscores that education systems can be effective only if they promote the health and well-being of every member of the school community, with the World Health Organization advocating for HPS to be implemented in every school (World Health Organization, 2021).

In Canada, the availability of health promotion interventions varies across schools and jurisdictions. While some school-based health promotion issues or topics are mandated by the government (e.g. sex education, oral health), school principals have a great deal of autonomy in deciding which health promotion interventions or programs to implement in their schools, depending on existing policies, guidelines, available resources and priorities of their school communities (Schwartz *et al.*, 2010; Storey *et al*., 2016; Riglea *et al*., 2022). Despite this variability, some commonly used strategies and approaches to school-based health promotion include: holding health promotion events, campaigns, and challenges; implementing school food programs; and providing before- and after-school opportunities for students to remain active (e.g. biking and intramural programs), among others.

The delivery of school-based health promotion initiatives can be drastically disrupted by unexpected school closures during public health emergencies or natural disasters, as was evident during the COVID-19 pandemic (Sormunen *et al.*, 2022). COVID-19 pandemic-related school closures have profoundly affected children’s lifestyle behaviours (healthy eating, physical activity, limited screen time, sleep) and mental well-being (Cost *et al*., 2022; *Peralta et al*., 2022), which are critical to children’s physical, social and emotional development (Macera, 2005). Indeed, only 5% of 5- to 11-year-olds in Canada and 0.6% of 12- to 17-year-olds adhered to recommended levels of PA and screen time early in the pandemic, while 71% met sleep recommendations (Moore *et al*., 2020). Low adherence was especially marked among children living in socioeconomically disadvantaged settings (Guerrero *et al*., 2020). Early evidence reported an increase in depression symptoms and psychological distress in school children during the first lockdown in the Spring 2020 (Bignardi *et al*., 2020; Cost *et al*., 2021), which seemed to be part of global trends (Santomauro *et al*., 2021). And while the World Health Organization has recently declared an end to the COVID-19 public health emergency (World Health Organization, 2023), climate change will likely accelerate the frequency of public health emergencies and natural disasters (e.g. poor air quality due to wildfires, extreme heat), resulting in school closures. For example, in June 2023, the City School District of the City of New York (commonly known as New York City Public Schools), which includes 1851 schools and serves more than 1 million students, shifted to remote learning due to wildfire smoke from Canada (O’Kane, 2023). For the USA as a whole, natural disasters induced by climate change are projected to total 2.24 million student school day closures per year (United States Environmental Protection Agency, 2023).

Schools play a critical role in the social lives of children and are an integral source of social support in their communities. During school closures, this critical function was disrupted (Loades *et al*., 2020). For children in disadvantaged settings, suspension of school-based programming (nutrition programs, learning and mental wellness support) heightens their vulnerability even further. Given the prospect of future school closures due to public health emergencies or natural disasters, it is imperative we consider the lessons from COVID-19 and put to use the innovative practices of delivering health promotion devised in response to COVID-19 pandemic-related school closures (e.g. sharing videos and readings, hosting online physical activity classes, using social media to foster connectedness between students and their teachers). However, education experts first need to agree on the best way forward and which health promotion practices should be included in school emergency preparedness plans. The objectives of this study were to characterize health promotion practices delivered during the COVID-19 pandemic-related school closures in Canadian elementary schools and to seek consensus among experts in the education sector on these practices in terms of their feasibility, acceptability, reach, effectiveness, cost-effectiveness and other criteria.

## METHODS

### Approach

First, to compile a comprehensive list of all health promotion activities delivered during the initial, prolonged COVID-19 pandemic-related school closure in Spring 2020, we reviewed the social media pages (e.g. Twitter, Facebook) of 87 schools across Alberta, Canada. We identified 60 health promotion activities and worked with our partners from the education sector APPLE Schools (A Project Promoting Healthy Living for Everyone in Schools) (Fung *et al*., 2012) to review this list for completeness and accuracy. In collaboration with our education partners, we grouped these activities into 15 health promotion practices based on the similarity and/or underlying concepts (Maximova *et al*., 2022) (Supplementary Table S1). Next, these practices were assigned to four broad categories: those that focus on raising awareness of healthy lifestyle behaviours and mental wellness, holding virtual events, providing tangible supports, and promoting school–student–family connectedness. We then employed the Delphi technique (Barrett and Heale, 2020; Niederberger and Spranger, 2020) with three rounds of online surveys (predefined closing criterion) administered to two expert panels. The unique feature of the Delphi technique is that it harnesses the collective wisdom of experts on a specific topic. Considering differences between schools in terms of their priorities and resources, judging health promotion practices on their feasibility, acceptability, reach, effectiveness, cost-effectiveness and other criteria requires taking into account a diverse set of expert opinions to reduce the risk of making uninformed decisions based on a single perspective.

### Recruitment and sampling

Expert panel 1 (EP1) consisted of practitioners (e.g. principals, teachers, school health champions) working with students and implementing health promotion activities in schools. Expert panel 2 (EP2) consisted of experts who are actively involved in planning (but not implementing) health promotion activities (e.g. health promotion leads and wellness coaches at the school board or provincial level, academics working in the field of health promotion research, school board representatives [e.g. superintendents, directors of student services]). To ensure that we have representatives from urban and rural, as well as North, Central and Southern parts of Alberta, Canada, we used different recruitment strategies. To recruit EP1 participants, we approached selected school boards and schools across Alberta and advertised the study during the Alberta Teachers Convention in February 2022. To recruit participants for EP2, we leveraged our professional networks. We also advertised the study in a purposive sample of researchers working in the field of health promotion and comprehensive school health in Alberta, Canada. In total, 51 prospective participants expressed their interest in the study, and 43 completed the Round 1 survey.

### Data collection

The surveys were pilot tested by two school health champions and one assistant superintendent to refine the content of the Delphi survey and ensure the readability of the health promotion practices and statements derived from an environmental scan on studies in which public health and clinical activities and practices were evaluated based on certain criteria. For each health promotion practice, EP1 participants were asked to rate their agreement on a 5-point Likert scale (strongly disagree, disagree, neither, agree, strongly agree) with the following statements: this practice (i) is easy and quick to implement; (ii) is cost-efficient to put in place; (iii) is effective in promoting health and wellness among students; (iv) is well received by most students; (v) is well received by teachers and school staff; (vi) is helping build and maintain healthy school culture; and (vii) can be used for years to come. EP2 participants were asked to rate their agreement with the following statements: this practice (i) reaches most students and families within a school; (ii) reaches the students and families who need it most; (iii) requires specific planning, preparation and training before implementation; (iv) requires additional staff time; (v) requires support or partnerships from outside the school; (vi) requires additional funding; (vii) is sustainable over time given staff turnover, costs, training, etc.; (viii) is easily integrated into the curriculum. Thus, in Round 1, EP1 and EP2 participants rated their agreement with 105 and 120 statements, respectively. In addition to rating the statements, participants were asked to justify their choice using open-ended comment boxes next to each statement, suggest additional practices if they perceived some were missing, and complete a short demographic form inquiring about their gender, age, region of residence, primary role, and how long they have been in this primary role.

In Rounds 2 and 3, surveys included aggregated quantitative results (but not individual participant responses) from the previous round presented as stacked bar charts for each statement with the frequencies of 5 response options. To accommodate colour-blind individuals, this information was also presented in text format. Participants who had not completed the survey in five days received a reminder to complete the survey in the next 2 days. The duration of each round was seven days, with 3 to 5 days between the rounds to analyse quantitative and qualitative data and incorporate the findings from the previous round into the next round. Three rounds of the survey in each expert panel were administered within six weeks. All participants were provided with compensation for their time, prorated depending on the number of rounds completed: $30 value electronic gift card if only Round 1 was completed, $60—if Round 1 and 2, and $90—if all three rounds. The *LimeSurvey* (Limesurvey GmbH, n.d.) platform was used to administer the Delphi survey. Health Research Ethics Board of the University of Alberta (Pro00116820) and Unity Health (REB#22-019) approved all procedures. All participants provided informed consent prior to the study.

### Data analysis

Descriptive statistics were used to describe the demographic characteristics of EP1 and EP2 participants. Frequencies of response options were calculated for each statement. Consensus was defined *a priori* as at least 70% of participants stating that they (strongly) agree, neither, or (strongly) disagree with a statement (i.e. a proportion within a range, unrestricted). While the stability of responses was not included in the consensus definition, we calculated percent agreement and Gwet’s AC1 (Gwet, 2008) to estimate inter-rater reliability. Statements for which consensus was reached were dropped from the subsequent round.

## RESULTS

In EP1, 23 of 29 individuals who expressed initial interest completed Round 1 (response rate [RR] 79.3%), and 22 completed Round 2 (RR 95.7%) and Round 3 (RR 100.0%), for the overall RR of 75.8%. In EP2, 20 of 22 who expressed initial interest completed Round 1 (RR 90.9%), 16 completed Round 2 (RR 80.0%), and 15 Round 3 (RR 93.8%), for the overall RR of 68.2%. Demographic characteristics of panel participants are provided in Table 1.

**Table 1:** Demographic information for the experts who participated in Round 1.

	Expert panel 1 (*n* = 23)	Expert panel 2 (*n* = 20)
Gender, %		
Female	87.0	85.0
Male	13.0	10.0
Other	0	5.0
Age, %		
18–24 years old	0	0
25–34 years old	30.4	15.0
35–44 years old	30.4	25.0
45–54 years old	30.4	50.0
55–64 years old	8.8	5.0
65 years and older	0	0
Prefer not to answer	0	5.0
Population centre^†^, %		
Rural	8.7	10.0
Small population centre	34.8	15.0
Medium population centre	26.1	15.0
Large population centre	30.4	60.0
Primary role, %		
Teacher	95.7	n/a
Principal	4.3	n/a
Health promotion professional	n/a	65.0
School board representative	n/a	30.0
Researcher	n/a	5.0
Experience in the primary role, %		
<1 year	0	0
1–4 years	8.7	25.0
5–9 years	26.1	30.0
10–15 years	13.0	10.0
>15 years	52.2	35.0

^†^Rural: <1000 people; Small population centre: 1000–29 999 people; Medium population centre: 30 000–99 999 people; Large population centre: >100 000 people.

**Fig. 1:**
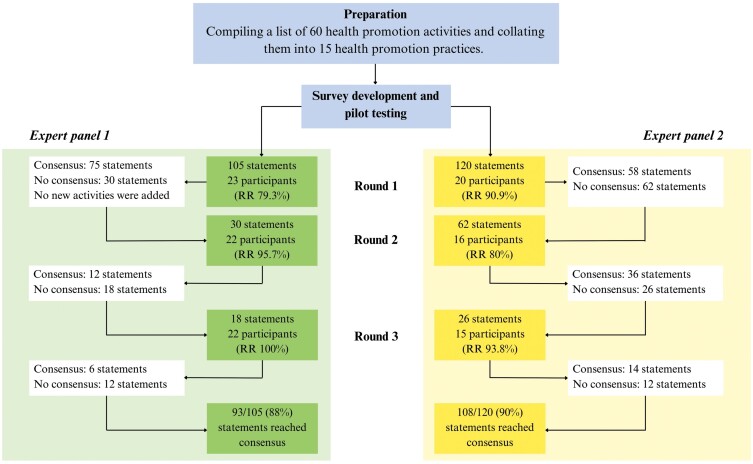
Flowchart of the Delphi process in two expert panels.

The flowchart for the Delphi process is presented in Figure 1. Consensus was reached for 93 of 105 and 108 out of 120 statements in EP1 and EP2, respectively. Participants were given the option to suggest additional health promotion practices: three practices were suggested but these were not substantially different from existing practices and therefore were not considered in the subsequent rounds.

Experts came to the consensus that, in general, practices that focus on raising awareness of healthy lifestyle behaviours and mental wellness are easy and quick to implement, effective and cost-effective, sustainable and easy to integrate into the curriculum, can be well received by students and teachers, have the potential to improve school culture, and do not require additional funding and resources (with the exception of practices #2 and #5) (Supplementary Table S2). However, practices #1 and #4 might not reach students who need them the most. All of these practices require planning, preparation and training before implementation and additional staff time, and most of them require support or partnerships from outside the school. Practices that focused on holding virtual events (i.e. holding virtual events, clubs and sessions) were rated similarly to the previous group of practices. Participants agreed that these practices might be effective in promoting health and wellness, well received by students (but not teachers and staff), sustainable and easy to integrate into the school curriculum. They might reach most students (but not those who need them the most) and help build and maintain a healthy school culture. However, these practices require planning, preparation and training, and additional funding and support from partners. The same considerations apply to practices focusing on providing tangible supports (either equipment to engage students in physical activity or food), but these practices have the potential to reach students needing these supports the most. The majority of participants noted that practices focusing on providing tangible support might not be easy and quick to implement, but they might be cost-effective. Practices that focus on promoting connectedness with students and families were rated similarly to practices that focus on raising awareness of healthy lifestyle behaviours and mental wellness, but unlike this first group of practices, they have the potential to reach those who need them the most.

As seen in Table 2, percent agreement varied substantially across statements, with as low as 31% (practice #1, statement ‘[this practice will be] used for years to come’ in Round 3) to 100%. Similar trends were observed for Gwet’s AC1 scores (Table 3), which varied from negative values indicating no agreement (e.g. AC1 = −0.03 for the statement ‘reach students and families who need it most’ for practice #2) to 1.00 indicating perfect agreement (e.g. ‘[strengthening health promotion messaging in lessons and curriculum] will be used for years to come’). For most statements that did not achieve consensus after Round 1, percent agreement and Gwet’s AC1 coefficients gradually increased in subsequent rounds. The following statements appeared particularly difficult to reach consensus on a practice is cost-effective, easy and quick to implement, will be used for years to come, and requires additional funding and resources.

**Table 2:** Percent agreement with each of the 15 statements for each of the 15 health promotion practices across three rounds of the Delphi survey

Statement^†^	Round	Practice category and number														
		Awareness of healthy lifestyle behaviours and mental wellness							Virtual events		Tangible supports		School–student–family connectedness			
		1	2	3	4	5	6	7	8	9	10	11	12	13	14	15
1	1	83	83	62	91	75	75	69	45	44	50	49	35	45	40	51
	2	–	–	–	–	–	–	–	48	48	41	41	46	49	40	83
	3	–	–	–	–	–	–	–	58	55	48	48	56	63	44	–
2	1	91	83	69	91	75	100	76	48	68	42	39	64	83	62	83
	2	–	–	–	–	–	–	–	41	–	41	42	–	–	–	–
	3	–	–	–	–	–	–	–	50	–	50	49	–	–	–	–
3	1	56	91	83	76	83	75	68	56	62	91	83	91	83	83	69
	2	–	–	–	–	–	–	–	–	–	–	–	–	–	–	–
	3	–	–	–	–	–	–	–	–	–	–	–	–	–	–	–
4	1	68	76	62	62	91	76	52	48	40	100	70	83	83	83	53
	2	–	–	–	–	–	–	52	61	56	–	–	–	–	–	83
	3	–	–	–	–	–	–	–	–	–	–	–	–	–	–	–
5	1	91	75	51	52	76	75	57	40	39	76	83	33	56	63	62
	2	–	–	91	83	–	–	–	45	50	–	–	34	–	–	–
	3	–	–	–	–	–	–	–	91	83	–	–	46	–	–	–
6	1	75	91	83	75	76	83	83	69	63	91	91	91	83	76	57
	2	–	–	–	–	–	–	–	–	–	–	–	–	–	–	–
	3	–	–	–	–	–	–	–	–	–	–	–	–	–	–	–
7	1	70	58	53	53	50	56	76	45	38	57	43	49	63	37	53
	2	–	–	82	74	50	83	–	32	45	–	32	45	–	39	74
	3	–	–	–	–	–	–	–	41	68	–	31	45	–	43	–
8	1	71	71	100	65	80	65	100	59	57	57	45	63	63	44	89
	2	–	–	–	–	–	–	–	–	–	–	77	–	–	88	–
	3	–	–	–	–	–	–	–	–	–	–	–	–	–	–	–
9	1	33	31	89	37	49	44	63	33	35	51	71	56	63	40	35
	2	68	37	–	66	68	36	–	60	66	66	–	–	–	66	46
	3	–	41	–	–	–	47	–	–	–	–	–	–	–	–	64
10	1	37	89	71	35	42	38	42	89	71	51	80	59	45	80	54
	2	77	–	–	34	68	43	42	–	–	51	–	–	60	–	68
	3	–	–	–	87	–	66	–	–	–	–	–	–	–	–	–
11	1	49	80	50	37	42	45	56	80	71	63	80	100	80	89	63
	2	88	–	77	58	60	58	–	–	–	–	–	–	–	–	–
	3	–	–	–	–	–	–	–	–	–	–	–	–	–	–	–
12	1	44	37	50	44	31	50	71	35	65	63	100	36	36	36	39
	2	38	43	66	43	31	58	–	–	–	–	–	58	68	38	88
	3	41	40	–	66	58	–	–	–	–	–	–	–	–	75	–
13	1	42	42	42	36	35	63	31	57	30	100	89	36	36	38	45
	2	66	48	42	51	36	–	34	–	38	–	–	77	66	33	66
	3	–	49	58	58	47	–	44	–	54	–	–	–	–	44	–
14	1	63	50	89	71	71	56	63	47	49	39	32	43	44	39	63
	2	–	58	–	–	–	–	–	57	51	51	50	48	68	60	–
	3	–	–	–	–	–	–	–	–	75	44	52	66	–	–	–
15	1	63	80	89	57	80	38	37	57	63	36	37	40	40	37	35
	2	–	–	–	–	–	39	88	–	–	77	33	46	77	33	43
	3	–	–	–	–	–	87	–	–	–	–	31	46	–	47	64

^†^Statements: A practice will 1) be easy and quick to implement; 2) be cost-efficient to put in place; 3) be effective in promoting health and wellness among students; 4) be well received by most students; 5) be well received by teachers and school staff; 6) be helping build and maintain healthy school culture; 7) be used for years to come; 8) reach most students and families within a school; 9) reach the students and families who need it most; 10) require specific planning, preparation and training before implementation; 11) require additional staff time; 12) require support or partnerships from outside the school; 13) require additional funding; 14) be sustainable over time given staff turnover, costs, training, etc.; 15) be easily integrated into the curriculum (Program of Studies).

**Table 3:** Gwet’s AC1 for 15 health promotion practices across three rounds of the Delphi surveys

Statement^†^	Round	Practice category and number														
		Awareness of healthy lifestyle behaviours and mental wellness							Virtual events		Tangible supports		School-student-family connectedness			
		1	2	3	4	5	6	7	8	9	10	11	12	13	14	15
1	1	0.82	0.82	0.54	0.91	0.72	0.72	0.63	0.25	0.23	0.35	0.32	0.06	0.25	0.16	0.36
	2	–	–	–	–	–	–	–	0.32	0.31	0.18	0.18	0.28	0.33	0.16	0.81
	3	–	–	–	–	–	–	–	0.48	0.42	0.31	0.31	0.45	0.55	0.24	–
2	1	0.91	0.82	0.63	0.91	0.72	1.00	0.73	0.30	0.63	0.20	0.13	0.57	0.82	0.54	0.82
	2	–	–	–	–	–	–	–	0.17	–	0.18	0.19	–	–	–	–
	3	–	–	–	–	–	–	–	0.35	–	0.35	0.33	–	–	–	–
3	1	0.44	0.91	0.82	0.73	0.82	0.72	0.63	0.44	0.54	0.91	0.82	0.91	0.82	0.82	0.63
	2	–	–	–	–	–	–	–	–	–	–	–	–	–	–	–
	3	–	–	–	–	–	–	–	–	–	–	–	–	–	–	–
4	1	0.63	0.73	0.54	0.54	0.91	0.73	0.37	0.3	0.16	1.00	0.65	0.82	0.82	0.82	0.40
	2	–	–	–	–	–	–	0.37	0.53	0.45	–	–	–	–	–	0.81
	3	–	–	–	–	–	–	–	–	–	–	–	–	–	–	–
5	1	0.91	0.72	0.36	0.37	0.73	0.72	0.45	0.16	0.14	0.73	0.82	0.01	0.44	0.55	0.54
	2	–	–	0.91	0.81	–	–	–	0.25	0.35	–	–	0.03	–	–	–
	3	–	–	–	–	–	–	–	0.91	0.81	–	–	0.27	–	–	–
6	1	0.72	0.91	0.82	0.72	0.73	0.82	0.82	0.63	0.55	0.91	0.91	0.91	0.82	0.73	0.45
	2	–	–	–	–	–	–	–	–	–	–	–	–	–	–	–
	3	–	–	–	–	–	–	–	–	–	–	–	–	–	–	–
7	1	0.65	0.47	0.40	0.40	0.34	0.44	0.73	0.25	0.13	0.45	0.22	0.32	0.55	0.09	0.40
	2	–	–	0.81	0.71	0.34	0.81	–	0.00	0.25	–	–0.01	0.25	–	0.14	0.71
	3	–	–	–	–	–	–	–	0.17	0.62	–	–0.03	0.25	–	0.22	–
8	1	0.66	0.66	1.00	0.58	0.77	0.58	1.00	0.49	0.46	0.46	0.26	0.55	0.55	0.24	0.89
	2	–	–	–	–	–	–	–	–	–	–	0.74	–	–	0.87	–
	3	–	–	–	–	–	–	–	–	–	–	–	–	–	–	–
9	1	0.03	–0.03	0.89	0.10	0.33	0.25	0.55	0.03	0.06	0.37	0.66	0.44	0.55	0.16	0.06
	2	0.62	0.10	–	0.59	0.62	0.08	–	0.51	0.59	0.59	–	–	–	0.59	0.27
	3	–	0.18	–	–	–	0.29	–	–	–	–	–	–	–	–	0.56
10	1	0.10	0.89	0.66	0.06	0.19	0.12	0.19	0.89	0.66	0.37	0.77	0.49	0.26	0.77	0.42
	2	0.74	–	–	0.05	0.62	0.21	0.19	–	–	0.37	–	–	0.51	–	0.62
	3	–	–	–	0.86	–	0.59	–	–	–	–	–	–	–	–	–
11	1	0.33	0.78	0.34	0.10	0.19	0.26	0.44	0.78	0.66	0.55	0.77	1.0	0.77	0.89	0.55
	2	0.87	–	0.74	0.47	0.51	0.47	–	–	–	–	–	–	–	–	–
	3	–	–	–	–	–	–	–	–	–	–	–	–	–	–	–
12	1	0.24	0.10	0.34	0.24	−0.03	0.34	0.66	0.46	0.58	0.55	1.00	0.07	0.07	0.07	0.15
	2	0.12	0.23	0.59	0.23	−0.02	0.47	–	–	–	–	–	0.47	0.62	0.12	0.87
	3	0.18	0.17	–	0.59	0.48	–	–	–	–	–	–	–	–	0.72	–
13	1	0.19	0.20	0.19	0.07	0.05	0.55	−0.03	0.46	−0.05	1.00	0.89	0.07	0.07	0.12	0.26
	2	0.59	0.30	0.23	0.36	0.08	–	0.05	–	0.12	–	–	0.74	0.59	0.01	0.59
	3	–	0.32	0.48	0.48	0.29	–	0.24	–	0.42	–	–	–	–	0.24	–
14	1	0.55	0.34	0.89	0.66	0.66	0.44	0.55	0.30	0.33	0.15	0.00	0.21	0.25	0.15	0.55
	2	–	0.47	–	–	–	–	–	0.46	0.36	0.36	0.35	0.30	0.62	0.51	–
	3	–	–	–	–	–	–	–	–	0.72	0.24	0.39	0.59	–	–	–
15	1	0.55	0.78	0.89	0.46	0.78	0.12	0.10	0.46	0.55	0.07	0.10	0.16	0.16	0.10	0.05
	2	–	–	–	–	–	0.15	0.87	–	–	0.74	0.03	0.27	0.74	0.03	0.21
	3	–	–	–	–	–	0.86	–	–	–	–	−0.01	0.27	-	0.29	0.56

^†^Statements: A practice will: 1) be easy and quick to implement; 2) be cost-efficient to put in place; 3) be effective in promoting health and wellness among students; 4) be well received by most students; 5) be well received by teachers and school staff; 6) be helping build and maintain healthy school culture; 7) be used for years to come; 8) reach most students and families within a school; 9) reach the students and families who need it most; 10) require specific planning, preparation and training before implementation; 11) require additional staff time; 12) require support or partnerships from outside the school; 13) require additional funding; 14) be sustainable over time given staff turnover, costs, training, etc.; 15) be easily integrated into the curriculum (Program of Studies).

## DISCUSSION

In this study, we synthesized the lessons from COVID-19 and revealed 15 health promotion practices that can be considered in school emergency preparedness plans and delivered during school closures. To equip school administrators and school boards with comprehensive information on each of these practices, we invited more than 40 experts working in the education sector to rate each practice in terms of criteria commonly used when making informed decisions about whether or not to implement an intervention in the real world. These practices are assigned to four categories: those that focus on raising awareness of healthy lifestyle behaviours and mental wellness, holding virtual events, providing tangible supports, and promoting school-student-family connectedness. This Delphi study showed that all practices require additional planning, preparation, training, and staff time, albeit likely to a different degree. Moreover, not all of them reach students and families who need them the most. Only those that focus on providing tangible supports (e.g. equipment, food) and promoting school–student–family connectedness on an individual basis can reach the most vulnerable populations. Lastly, while most of the considered practices require support and partnership outside of the school community, not all require additional funding (e.g. practices that focus on raising awareness of healthy lifestyle behaviours and mental wellness and promoting school-student-family connectedness).

To our knowledge, this study is the first to identify health promotion practices that can be delivered during school closures and to seek consensus among education experts on their utility. Practical guidance on delivering health promotion during alternate learning is urgently needed, given the future prospects of schools being temporarily closed during public health emergencies or natural disasters. These practices may also apply to other remote learning situations: for example, for rural and remote locations where regular school attendance is not always possible; for children who are homeschooled on a brief (e.g. feeling unwell) or regular basis.

While all health promotion practices were rated by the experts as effective, implementation efforts must recognize the local context of each school, with its financial and human resource constraints, unique needs and priorities (Herlitz *et al*., 2020). Indeed, previous research suggests that transitioning health promotion programming to remote learning is riddled with challenges (Camp-Spivey *et al.*, 2021). In their mixed-methods study, Camp-Spivery *et al*. surveyed and interviewed 1311 and 28 school administrators, respectively, on how school-based interventions were affected by COVID-19 pandemic-related school closures. Participants emphasized the need for capacity building to equip teachers and school staff with a roster of activities that can be implemented during school closures. Participants reported that the delivery of health promotion programming was on the back burner during school closures, while academic content areas (e.g. math, reading, science) were prioritized. Moreover, being unable to observe students engaging in health promotion activities (e.g. virtual physical education class) was also a challenge, as this prevented teachers from assessing students’ engagement in these activities and delivering them to their full extent and potential. Despite these challenges, there was unanimity among participants about the need to continue the delivery of health-promoting programming targeting lifestyle behaviours during school closures. Yet, as our study shows, implementing all 15 health practices requires additional planning, preparation and training. Therefore, school authorities and jurisdictions should consider investments in implementation support specialists (e.g. health promotion facilitators) who can tailor health promotion programming to specific school contexts and help build health promotion capacity among school staff.

When choosing health promotion practices to be delivered during school closures, an important consideration pertains to equity. School closures tend to reveal and amplify flagrant social inequalities. Since schools and families have very different resources available to them, Alexander and Sharek (2021) write that schools in high-income neighbourhoods tended to adapt to remote learning during the COVID-19 pandemic-related school closures much faster than schools with limited resources and access to devices and the Internet. However, apart from differences in access to devices and the Internet that limit the participation of students and families living in lower socioeconomic neighbourhoods in learning and health promotion activities delivered online, these vulnerable populations often have limited access to safe outdoor spaces. This might prevent children and youth from engaging in physical activities if there are no favourable conditions to do these activities at home. These inequalities put students from low socioeconomic backgrounds at an increased risk of having worse physical health and mental well-being, but also at an increased risk of social exclusion and isolation since participation in virtual activities might be one of the avenues for socializing with peers (Alexander and Shareck, 2021). Therefore, with respect to equity, health promotion practices that focus on raising awareness through virtual means and on holding virtual events might not be those of choice, particularly in schools that serve diverse populations of students and those located in socioeconomically disadvantaged areas.

Another consideration for the practices focusing on raising awareness through virtual means is well articulated by Lorenc and Oliver (2014). It is a mistake to assume that providing families with informational material is enough and that families would eagerly implement these activities at home. Instead, this practice shifts the responsibility of delivering health promotion to parents, which, at a time of upheaval (either caused by a pandemic, natural disaster or other calamities), adds unnecessary stress to and expectations from parents. In our previous qualitative study on parents’ perspectives on pandemic-related changes to health promotion programming, very few parents reported opening emails with informational material, while most of the parents were too overwhelmed and did not access them, finding these materials intrusive, being preoccupied with other things, and facing their own personal stressors (Dabravolskaj *et al*., 2023).

This study has important strengths. We recruited a panel of 43 experts, with more than 70% of participants having five or more years of experience in planning and/or delivering health promotion programming in schools. The inventory of practices comes from school social media pages of a large selection of schools. However, there are several limitations to consider when interpreting the results of this study. Since APPLE Schools intervention targets schools located in socioeconomically disadvantaged rural and remote areas, it might be that a broader selection of schools would reveal additional practices, albeit including urban schools might not help identify practices suitable for disadvantaged children and families with limited access to resources and technology. Finally, as emphasized by the Organization for Economic Co-operation and Development (2020), it is essential to engage youth and their sense of agency to help co-create and implement new and tailor existing health promotion practices, among other policy responses and recovery plans. Therefore, future research in this area would benefit from gathering students’ perspectives on which health promotion practices appeal to them.

## CONCLUSION

This study provides a comprehensive overview of health promotion practices that can improve children’s lifestyle behaviours and mental wellness during prolonged school closures due to pandemic lockdowns, evacuations resulting from wildfires and flooding and other unforeseen events leading to school closures. Work is currently underway to translate these findings into a comprehensive, practical, actionable and scalable online toolkit with an array of health promotion practices that schools can choose from (depending on local resources and unique context) to inform emergency preparedness plans, build capacity among educators to support children’s active living and mental well-being during alternate learning, and ameliorate health inequalities that are often exacerbated by public health emergencies and other natural disasters.

## Supplementary Material

daad172_suppl_Supplementary_Tables_S1-S2
