# Supplementary material for: Delivering health promotion during school closures in public health emergencies: building consensus among Canadian experts
Source: Health Promot Int. 2023 Dec 13;38(6):daad172. doi: 10.1093/heapro/daad172 (PMC10716908; doi:10.1093/heapro/daad172)
Supplement: daad172_suppl_Supplementary_Tables_S1-S2 [file daad172_suppl_supplementary_tables_s1-s2.docx]

**Supplementary materials**

Table S1. Health promotion practices implemented during school closures in Spring 2020.

| **Practice** | **Description** |
| --- | --- |
| ***Raising awareness of healthy lifestyle behaviours and mental wellness*** | |
| 1 | Using school social media pages, newsletters or other communication channels to share existing resources that promote active, healthy living and wellness (e.g., wellness websites, mindfulness or physical activity ideas to try at home, recipe ideas, information on hot topics or questions coming from the school community). |
| 2 | Holding wellness challenges and campaigns or sharing announcements (e.g., made by school administration) that promote active, healthy living and wellness. |
| 3 | Strengthening health promotion messaging in lessons/curriculum. |
| 4 | Encouraging students to engage in a variety of outdoor activities in their free time via school social media pages, newsletters, or other communication channels. |
| 5 | Encouraging students to engage in self-expression activities (creating and displaying signs with positive messages (e.g., on school fences, students’ front lawns), window art, leaving positive messages in chalk on sidewalks, etc.) to promote wellness. |
| 6 | Leaving celebratory posts or videos on school social media pages or other communication channels to promote wellness. |
| 7 | Raising awareness among families of available school or community-based mental health support. |
| ***Holding virtual events*** | |
| 8 | Holding live virtual events that promote healthy lifestyle behaviours and mental wellness. |
| 9 | Holding virtual clubs and sessions that focus specifically on promoting mental health and wellness. |
| ***Providing tangible supports*** | |
| 10 | Distributing equipment (e.g., physical activity equipment (e.g., skipping ropes, bubbles, soccer balls) or family games (e.g., board, card, word games) to help students engage in active, healthy living and wellness activities at home. |
| 11 | Providing tangible food-based supports (e.g., distributing gift cards for grocery shopping, making food hampers or some food items available for families to pick up, as well as partnering with local restaurants for additional food-based support). |
| ***Promoting school-student-family connectedness*** | |
| 12 | Connecting with all students one-on-one by sending personal messages or giving a call to chat about their lives, what they are doing outside of schoolwork, what they are struggling with, collecting feedback on their needs and how teachers might help. |
| 13 | Connecting with families to discuss how they are doing, share positive or encouraging messages, inquire whether additional school support is required. |
| 14 | Organizing parades, drive-by’s or drive-thru’s to see students in-person (at a distance, if needed) and maintain connection to the school community. |
| 15 | Collecting feedback from families to gain insight into their perspective of alternate learning, required school supports, among other things. |

Table S2. Percent of participants who (strongly) disagreed v. (strongly) agreed with each of the 15 statements for each of the 15 health promotion practices.

| **Statement^†^** | **Practice category and number** | | | | | | | | | | | | | | |
| --- | --- | --- | --- | --- | --- | --- | --- | --- | --- | --- | --- | --- | --- | --- | --- |
|  | **Awareness of healthy lifestyle behaviours and mental wellness** | | | | | | | **Virtual events** | | **Tangible supports** | | **School-student-family connectedness** | | | |
|  | **1** | **2** | **3** | **4** | **5** | **6** | **7** | **8** | **9** | **10** | **11** | **12** | **13** | **14** | **15** |
| 1 | 9 v. 91 | 4 v. 91 | 9 v. 78 | 0 v. 96 | 9 v. 87 | 9 v. 87 | 13 v. 83 | 27 v. 73 | 68 v. 32 | 18 v. 68 | 55 v. 45 | 23 v. 73 | 77 v. 23 | 41 v. 55 | 9 v. 91 |
| 2 | 4 v. 96 | 4 v. 91 | 4 v. 83 | 4 v. 96 | 9 v. 87 | 0 v. 100 | 13 v. 87 | 68 v. 23 | 9 v. 83 | 23 v. 23 | 18 v. 68 | 0 v. 78 | 4 v. 91 | 13 v. 78 | 0 v. 91 |
| 3 | 13 v. 74 | 4 v. 96 | 9 v. 91 | 13 v. 87 | 0 v. 91 | 4 v. 87 | 9 v. 83 | 13 v. 74 | 13 v. 78 | 0 v. 96 | 4 v. 91 | 0 v. 96 | 4 v. 91 | 0 v. 91 | 4 v. 83 |
| 4 | 9 v. 83 | 13 v. 87 | 9 v. 78 | 13 v. 78 | 4 v. 96 | 0 v. 87 | 0 v. 100 | 18 v. 77 | 23 v. 73 | 0 v. 100 | 0 v. 83 | 0 v. 91 | 4 v. 91 | 0 v. 91 | 0 v. 91 |
| 5 | 0 v. 96 | 9 v. 87 | 0 v. 95 | 9 v. 91 | 0 v. 87 | 4 v. 87 | 17 v. 74 | 95 v. 0 | 91 v. 9 | 0 v. 87 | 0 v. 91 | 59 v. 36 | 13 v. 74 | 4 v. 78 | 13 v. 78 |
| 6 | 4 v. 87 | 0 v. 96 | 4 v. 91 | 4 v. 87 | 0 v. 87 | 9 v. 91 | 0 v. 91 | 4 v. 83 | 4 v. 78 | 0 v. 96 | 4 v. 96 | 0 v. 96 | 0 v. 91 | 0 v. 87 | 9 v. 74 |
| 7 | 0 v. 83 | 4 v. 74 | 5 v. 91 | 5 v. 86 | 0 v. 100 | 9 v. 91 | 0 v. 87 | 59 v. 18 | 82 v. 14 | 9 v. 74 | 41 v. 32 | 64 v. 18 | 4 v. 78 | 59 v. 32 | 5 v. 86 |
| 8 | 11 v. 84 | 11 v. 84 | 0 v. 100 | 21 v. 79 | 5 v. 89 | 21 v. 79 | 0 v. 100 | 26 v. 74 | 21 v. 74 | 21 v. 74 | 13 v. 88 | 16 v. 79 | 11 v. 79 | 6 v. 94 | 5 v. 95 |
| 9 | 81 v. 19 | 53 v. 40 | 0 v. 95 | 81 v. 13 | 19 v. 81 | 53 v. 47 | 5 v. 79 | 75 v. 25 | 81 v. 13 | 13 v. 81 | 11 v. 84 | 16 v. 74 | 16 v. 79 | 13 v. 81 | 13 v. 80 |
| 10 | 13 v. 88 | 5 v. 95 | 11 v. 84 | 7 v. 93 | 19 v. 81 | 20 v. 80 | 0 v. 100 | 5 v. 95 | 11 v. 84 | 0 v. 100 | 5 v. 89 | 26 v. 74 | 25 v. 75 | 5 v. 89 | 19 v. 81 |
| 11 | 6 v. 94 | 0 v. 89 | 13 v. 88 | 19 v. 75 | 25 v. 75 | 19 v. 75 | 16 v. 74 | 11 v. 89 | 11 v. 84 | 16 v. 79 | 5 v. 89 | 0 v. 100 | 5 v. 89 | 5 v. 95 | 11 v. 79 |
| 12 | 40 v. 53 | 20 v. 60 | 13 v. 81 | 20 v. 80 | 73 v. 27 | 75 v. 19 | 5 v. 84 | 5 v. 74 | 0 v. 79 | 5 v. 79 | 0 v. 100 | 75 v. 19 | 81 v. 19 | 13 v. 87 | 94 v. 0 |
| 13 | 81 v. 13 | 27 v. 67 | 73 v. 27 | 73 v. 27 | 20 v. 67 | 79 v. 11 | 60 v. 33 | 21 v. 74 | 73 v. 13 | 0 v. 100 | 5 v. 95 | 88 v. 13 | 81 v. 13 | 33 v. 60 | 81 v. 6 |
| 14 | 11 v. 79 | 19 v. 75 | 5 v. 95 | 5 v. 84 | 5 v. 84 | 16 v. 74 | 16 v. 79 | 13 v. 75 | 13 v. 87 | 33 v. 60 | 67 v. 33 | 20 v. 80 | 19 v. 81 | 25 v. 75 | 16 v. 79 |
| 15 | 11 v. 79 | 0 v. 89 | 5 v. 95 | 21 v. 74 | 0 v. 89 | 0 v. 93 | 6 v. 94 | 5 v. 74 | 11 v. 79 | 0 v. 88 | 27 v. 27 | 0 v. 100 | 0 v. 88 | 13 v. 20 | 7 v. 80 |

^⸰^Consensus was defined as at least 70% of participants stating that they (strongly) agree, neither, or (strongly) disagree with a statement.

**^†^**Statements: A practice will: 1) be easy and quick to implement; 2) be cost-efficient to put in place; 3) be effective in promoting health and wellness among students; 4) be well received by most students; 5) be well received by teachers and school staff; 6) be helping build and maintain healthy school culture; 7) be used for years to come; 8) reach most students and families within a school; 9) reach the students and families who need it most; 10) require specific planning, preparation and training before implementation; 11) require additional staff time; 12) require support or partnerships from outside the school; 13) require additional funding; 14) be sustainable over time given staff turnover, costs, training, etc.; 15) be easily integrated into the curriculum (Program of Studies).

^⁕^Statements that did not achieve consensus after three rounds of the Delphi survey are shaded in grey.
